# Differential Impact of Cytochrome 2C19 Allelic Variants on Three Different Platelet Function Tests in Clopidogrel-Treated Patients

**DOI:** 10.3390/jcm10173992

**Published:** 2021-09-03

**Authors:** Renske H. Olie, Rachelle R. K. Hensgens, Petal A. H. M. Wijnen, Leo F. Veenstra, Bianca T. A. de Greef, Minka J. A. Vries, Paola E. J. van der Meijden, Jurriën M. ten Berg, Hugo ten Cate, Otto Bekers, Yvonne M. C. Henskens

**Affiliations:** 1 Department of Internal Medicine, Maastricht University Medical Center+ (MUMC+), 6229 HX Maastricht, The Netherlands; Rachelle.hensgens@hotmail.com (R.R.K.H.); h.tencate@maastrichtuniversity.nl (H.t.C.); 2 Laboratory for Clinical Thrombosis and Haemostasis, Cardiovascular Research Institute Maastricht (CARIM), Maastricht University, 6200 MD Maastricht, The Netherlands; minka.vries@hotmail.com (M.J.A.V.); p.vandermeijden@maastrichtuniversity.nl (P.E.J.v.d.M.); yvonne.henskens@mumc.nl (Y.M.C.H.); 3 Thrombosis Expertise Center, Maastricht University Medical Center+ (MUMC+), 6229 HX Maastricht, The Netherlands; 4 Central Diagnostic Laboratory, Maastricht University Medical Center+ (MUMC+), 6229 HX Maastricht, The Netherlands; petal.wijnen@mumc.nl (P.A.H.M.W.); o.bekers@mumc.nl (O.B.); 5 Department of Cardiology, Maastricht University Medical Center+ (MUMC+), 6229 HX Maastricht, The Netherlands; l.veenstra@mumc.nl (L.F.V.); jurtenberg@gmail.com (J.M.t.B.); 6 Department of Clinical Epidemiology and Medical Technology Assessment, Maastricht University Medical Center+ (MUMC+), 6229 HX Maastricht, The Netherlands; bianca.greef@mumc.nl; 7 Department of Cardiology, St. Antonius Hospital, 3435 CM Nieuwegein, The Netherlands

**Keywords:** pharmacogenetics, clopidogrel, platelet activation, platelet function test, *CYP2C19*

## Abstract

On-treatment platelet reactivity in clopidogrel-treated patients can be measured with several platelet function tests (PFTs). However, the agreement between different PFTs is only slight to moderate. Polymorphisms of the *CYP2C19* gene have an impact on the metabolization of clopidogrel and, thereby, have an impact on on-treatment platelet reactivity. The aim of the current study is to evaluate the differential effects of the *CYP2C19* genotype on three different PFTs. Methods: From a prospective cohort study, we included patients treated with clopidogrel following percutaneous coronary intervention (PCI). One month after PCI, we simultaneously performed three different PFTs; light transmission aggregometry (LTA), VerifyNow P2Y12, and Multiplate. In whole EDTA blood, genotyping of the *CYP2C19* polymorphisms was performed. Results: We included 308 patients treated with clopidogrel in combination with aspirin (69.5%) and/or anticoagulants (33.8%) and, based on *CYP2C19* genotyping, classified them as either extensive (36.4%), rapid (34.7%), intermediate (26.0%), or poor metabolizers (2.9%). On-treatment platelet reactivity as measured by LTA and VerifyNow is significantly affected by *CYP2C19* metabolizer status (*p* < 0.01); as metabolizer status changes from rapid, via extensive and intermediate, to poor, the mean platelet reactivity increases accordingly (*p* < 0.01). On the contrary, for Multiplate, no such ordering of metabolizer groups was found (*p* = 0.10). Conclusions: For VerifyNow and LTA, the on-treatment platelet reactivity in clopidogrel-treated patients correlates well with the underlying *CYP2C19* polymorphism. For Multiplate, no major effect of genetic background could be shown, and effects of other (patient-related) variables prevail. Thus, besides differences in test principles and the influence of patient-related factors, the disagreement between PFTs is partly explained by differential effects of the *CYP2C19* genotype.

## 1. Introduction

Dual antiplatelet therapy with aspirin and a P2Y12 receptor blocker (clopidogrel, prasugrel, or ticagrelor) is the cornerstone of antithrombotic therapy preventing recurrent cardiovascular events in patients undergoing percutaneous coronary intervention (PCI) [1]. Although guidelines favor more potent next-generation P2Y12 inhibitors, such as ticagrelor and prasugrel, in acute coronary syndrome (ACS) patients [1,2], clopidogrel remains the most widely prescribed P2Y12 inhibitor, partly due to clinical and economic factors [3]. There is substantial variability in pharmacodynamic action of clopidogrel, which translates into variation in clopidogrel effectiveness after PCI [4,5]. Besides several contributing clinical factors, this variability is partly explained by genetic polymorphisms encoding cytochrome P450 (CYP) 2C19, the hepatic enzyme involved in biotransformation of the prodrug clopidogrel to its active metabolite [6]. The hepatic conversion of clopidogrel to its active metabolite is a two-step biotransformation process. Because 85% of the prodrug clopidogrel is inactivated by esterases after intestinal absorption, only 15% is available for transformation to the active metabolite, for which the hepatic *CYP2C19* enzyme is a key determinant. The gene that codes the *CYP2C19* enzyme is highly polymorphic, and over 30 gene alleles have been identified [7]. The CYP2C19*1 allele is the most prevalent and represents a normal activity allele. CYP2C19*2 and CYP2C19*3 alleles are the most frequently observed polymorphisms leading to a complete loss of enzyme activity, with reduced clopidogrel conversion. This results in higher residual platelet reactivity [5,8,9,10] and is associated with a higher incidence of major adverse cardiovascular and cerebrovascular events [4,5,6,9,10,11]. On the other hand, the CYP2C19*17 allelic variant represents a gain-of-function mutation that leads to increased catalytic activity and increased production of active metabolites, which might result in more pronounced platelet inhibition, higher bleeding risk, and lower risk for ischemic events with clopidogrel [12,13,14,15]. However, other studies have reported no significant association between the *17 allele and ischemic and bleeding outcomes after accounting for the *2 allele, so its clinical relevance is still controversial [16,17]. In contrast to clopidogrel, the *CYP2C19* genotype does not impact the clinical effects of prasugrel or ticagrelor, for which clinical trials have shown the superiority over clopidogrel in reducing ischemic events, although accompanied by higher bleeding risks [18,19].

Especially in patients with multiple clinical risk factors, clinicians will have to weigh the bleeding and ischemic risks to individualize treatment decisions following PCI [20]. Platelet function tests (PFTs) could guide such decisions; however, although personalized treatment strategies based on PFTs have been evaluated, all have failed to show significant clinical benefit [21,22,23]. Moreover, multiple PFTs using different techniques are available, but previous studies have shown that the agreement between different PFTs is only slight to moderate, leading to conflicting results [24,25,26,27,28]. More recently, studies evaluating pharmacogenomic-based tailoring of P2Y12 inhibitors seemed to be more promising [29,30,31,32].

The aim of the current study is to evaluate the differential effects of *CYP2C19* genotypes on three different platelet function tests. Understanding the relationship between PFTs and genetic polymorphisms is important in the interpretation of the disagreement between these PFTs and determination of optimal antiplatelet therapy in high-risk patients, and it could help to explain the disappointing PFT-tailoring studies in comparison to the more promising pharmacogenomic approach.

## 2. Materials and Methods

This prospective cohort study is conducted at the Thrombosis Expertise Centre in the Maastricht University Medical Center (MUMC+) in the Netherlands. The study was reviewed and approved by the medical ethical committee of the MUMC+ (NL38767.068.11, METC number 11-2-096), and all patients provided written informed consent.

### 2.1. Study Population

Patients were selected from a cohort of high-risk patients with dual or triple antithrombotic therapy after PCI. This prospective cohort study is extensively described elsewhere [33]. In brief, all patients underwent PCI (either elective or following ACS) and were classified as high-risk patients by the presence of ≥3 predefined risk factors (Table A1). Patients either had dual antithrombotic therapy (a P2Y12 inhibitor plus aspirin or anticoagulants) or triple therapy (a combination of a P2Y12 inhibitor, aspirin, and anticoagulants) in the case of concomitant atrial fibrillation. For the current research question, we only selected the patients on clopidogrel for whom *CYP2C19* analysis had been performed by June 2018. Exclusion criteria were previously diagnosed platelet function disorders, a new ischemic event or coronary revascularization procedure ≤7 days, confirmed noncompliance, or signs of active infection.

### 2.2. Laboratory Measurements

On-treatment platelet reactivity was measured using three different PFTs. Blood for all these PFTs was drawn simultaneously 1–2 months after PCI, together with the sample needed for genotyping of the *CYP2C19* polymorphisms. Furthermore, we measured blood count and renal function.

#### 2.2.1. Multiple Electrode Impedance Aggregometry by Multiplate

Blood was collected in Hirudin Blood Tubes (3 mL, Double Wall; Verum Diagnostica GmbH, Munich, Germany). Adenosine diphosphate (ADP, 6.4 uM)-induced platelet aggregation was measured by the Multiplate analyzer (Dynabyte, Munich, Germany), according to the manufacturer’s instructions. The Multiplate analyzer is a multiple electrode impedance aggregometer that measures platelet aggregation in whole blood. Results are expressed as arbitrary aggregation units (AU).

#### 2.2.2. VerifyNow

Blood was collected in 3.2% sodium citrate Vacuette partial-fill tubes (2 mL; Greiner Bio-One, GmbH, Kremsmuntster, Austria). VerifyNow P2Y12 assay (Accumetrics Inc, San Diego, CA, USA) was performed according to the manufacturer’s instructions. This is a turbidimetric-based optical detection system, in which whole blood is added to a device using fibrinogen-coated microbeats. After addition of an agonist (20 µmol/L ADP–22 nmol/L prostaglandin E1), the increase in light transmittance is detected to measure platelet agglutination, and results are expressed in P2Y12 reaction units (PRU).

#### 2.2.3. Light Transmission Aggregometry (LTA)

Blood was collected in 3.2% sodium citrate Vacuette tubes (9 mL; Greiner Bio-One). For preparation of platelet-rich plasma, blood was centrifuged at 170× *g* for 10 min at 18 °C. For preparation of platelet-poor plasma, citrated blood was centrifuged at 2500× *g* for 5 min and then at 10,000× *g* for 10 min at 18 °C. Platelet count in platelet-rich plasma was adjusted with autologous platelet-poor plasma to 250 × 10^9^ platelets/L. ADP (20 μmol/L, Chrono-Par, CH 384) was added at 37 °C. The main result was the percentage of maximal platelet aggregation.

#### 2.2.4. *CYP2C19* Analysis

From whole EDTA blood, genomic DNA for genetic analysis was isolated with the MagNa Pure 96 DNA isolation system (Roche Diagnostics, Mannheim, Germany) according to the manufacturer’s instructions. Using the LightCycler^®^ (Roche Diagnostics), genotyping of the *CYP2C19* polymorphism was performed using a FRET LightMix^®^ assay (TIB MOLBIOL, Berlin, Germany). Genotypes were determined blinded without knowledge of platelet aggregation values. CYP variants were grouped according to the guideline of the Clinical Pharmacogenetic Implementation Consortium (CPIC) [8,34]; CYP2C19 *1/*1 is referred to as normal or extensive metabolizer (EM), genotypes with one loss-of function allele (CYP2C19 *1/*2, *1/*3 or *2/*17) were assembled under the term ‘intermediate metabolizer’ (IM), and the genotype with two loss-of-function alleles (CYP2C19 *2/*2) is referred to as ‘poor metabolizer’ (PM). The last group consists of the genotypes CYP2C19 *1/*17 and *17/*17, and is referred to as ‘rapid metabolizers’ (RM).

### 2.3. Statistical Analysis

Continuous variables are expressed as either mean with standard deviation (SD) for normally distributed traits or median with interquartile range (IQR) otherwise. Categorical variables are expressed as counts and percentages. The distribution of the data was estimated using visual inspection of histograms and confirmed using the Shapiro–Wilk test. To compare categorical variables between the metabolizer groups, the Chi-square test was used, with Fisher’s Exact Test when applicable. Normally distributed continuous variables were compared between metabolizer groups using ANOVA. The Jonckheere–Terpstra test for ordered alternatives was used to test for statistically significant ordinal trends between the metabolizer groups and for pairwise comparison of the different metabolizer groups compared to extensive metabolizers (reference category), with Bonferroni post-test to correct for multiple testing. Multivariable linear regression analysis was performed to adjust for other factors influencing platelet reactivity. Variables were included in the model based on existing literature or if univariate analysis indicated the variable to be associated with at least one of the platelet function tests at *p* < 0.20. Presence of multicollinearity was checked using variation inflation factors. Simple correlation between the three platelet function tests was assessed with Pearson correlation coefficients (ρ). A value of *p* < 0.05 was considered to be statistically significant. Statistical analyses were performed with IBM SPSS statistics version 25.0 and GraphPad Prism version 5.

## 3. Results

### 3.1. Baseline Characteristics

From the total cohort of 524 patients [33], 308 patients met the inclusion criteria and were included in this analysis. Baseline characteristics of the study population are shown in Table 1. Mean age is 75.2 (8.5) years, with 41.2% being female. All patients were treated with clopidogrel (100%) in combination with aspirin (69.5%) and/or anticoagulants (33.8%).

Table 2 shows the distribution of *CYP2C19* polymorphisms in our population, with 36.4% extensive (normal) metabolizers (*1/*1). About 34.7% of our population was classified as rapid metabolizers (*1/*17 or *17/*17), whereas 26.0% was intermediate metabolizers (*1/*2, *2/*17 or *1/*3), and 9 patients (2.9%) turned out to be poor metabolizers (*2/*2).

### 3.2. Agreement between Platelet Function Tests

All 3 platelet function tests were measured simultaneously after a median of 46 (37–59) days post-PCI. Simple correlation between platelet reactivity values was moderate for the 3 platelet function tests: ρ = 0.566 (*p* < 0.0001) for the correlFation between VerifyNow and LTA, ρ = 0.493 (*p* < 0.0001) for VerifyNow and Multiplate, and ρ = 0.423 (*p* < 0.0001) for LTA and Multiplate.

### 3.3. Residual Platelet Reactivity per Group of CYP2C19 Metabolism

**Figure 1:**
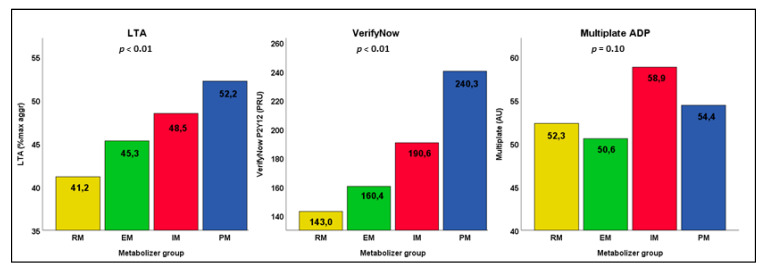
Mean platelet reactivity as measured by three platelet function tests in the clopidogrel group expressed per group of *CYP2C19* metabolism. Differences in platelet reactivity per group of *CYP2C19* metabolism were measured using ANOVA. Poor metabolizers (PM) have genotype CYP2C19*2/*2, intermediate metabolizers (IM) CYP2C19*1/*2, *1/*3, or *2/*17, extensive metabolizers (EM) CYP2C19*1/*1, and rapid metabolizers (RM) CYP2C19*1/*17 or *17/*17.

Mean values of residual platelet reactivity per group of *CYP2C19* metabolism as measured by LTA VerifyNow and Multiplate are shown in Figure 1. For LTA, the intermediate and poor metabolizers have higher residual platelet reactivity as compared to extensive metabolizers, whereas the rapid metabolizers have the lowest on-treatment platelet reactivity. The residual platelet reactivity as measured by the LTA is significantly affected by *CYP2C19* metabolizer status (*p* < 0.01). A Jonckheere–Terpstra test for ordered alternatives showed that there was a statistically significant trend of higher platelet reactivity with consecutive metabolizer groups; as metabolizer status changes from rapid, via extensive and intermediate, to poor, the mean LTA value increases accordingly (*p* < 0.01) (Table 3).

The same applies to the VerifyNow test; residual platelet reactivity is significantly affected by *CYP2C19* metabolizer status (*p* < 0.01), with a statistically significant trend of higher platelet reactivity when metabolizer status changes from rapid, extensive, intermediate to poor metabolizer (*p* < 0.01).

Contrary to the LTA and VerifyNow, for the Multiplate no such trend can be found; mean residual platelet reactivity is not significantly different (*p* = 0.10) between the metabolizer groups. Additionally, the Jonckheere–Terpstra test showed no statistically significant ordering of the metabolizer groups (*p* = 0.10).

### 3.4. Effect of Metabolizer Status on Platelet Reactivity

**Figure 2:**
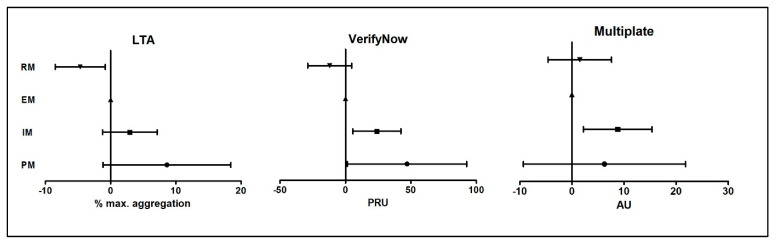
Different metabolizer groups as predictor of residual platelet reactivity in multivariate linear regression analysis. Partial regression coefficients (B) with 95% confidence interval for metabolizer group as predictor of residual platelet reactivity as measured by the 3 platelet function tests. Extensive metabolizers (EM) are the reference group. Abbreviations: PM, poor metabolizer; IM, intermediate metabolizer; EM, extensive metabolizer; RM, rapid metabolize. Max. aggregation, percentage of maximum aggregation; PRU, P2Y12 reaction units; AU, aggregation units.

Figure 2 shows the effect of the different metabolizer groups on the residual platelet reactivity as measured by LTA, VerifyNow, and Multiplate. Platelet reactivity in this multivariable model was adjusted for age, body weight, diabetes, renal insufficiency, previous stroke, current smoking, concomitant use of (es-)omeprazole, hemoglobin, platelet count, and use of aspirin and/or anticoagulants, based on results of the univariate analysis *(*Table A2*).* In this adjusted model, poor metabolizer status is associated with a nonsignificant increase of 8.8% in maximal aggregation in the LTA (Beta: 8.8; 95% CI: −1.0–18.6; *p* = 0.08) as compared to the EM group, whereas a rapid metabolizer status will lead to a decrease of 4.6% (Beta: −4.6; 95% CI: −8.5–−0.8; *p* = 0.02) in maximum aggregation of LTA. Compared to the wild type (EM), poor and intermediate metabolizer status is associated with an increase of 48.3 PRU (Beta: 48.3; 95% CI: 2.4–94.3; *p* = 0.04) and 22.5 PRU (Beta: 22.5; 95% CI: 4.0–40.9; *p* = 0.02) in VerifyNow, respectively (Table A2). As can be appreciated from Figure 2, the metabolizer status affects platelet reactivity as measured by both LTA and VerifyNow following an ordinal order; PM and IM status is associated with a (numerical) platelet reactivity increase and RM status with a (numerical) decrease. However, no such ordinal order could be found for the association of metabolizer status with platelet reactivity as measured by Multiplate.

### 3.5. Relative Importance of Metabolizer Status on Platelet Reactivity

Besides this effect of metabolizer status on differences in platelet reactivity between the PFTs, these differences are also explained by several clinical risk factors. Because the regression coefficients of all variables in the multivariable model are expressed in different measurement units, direct comparison of variables is difficult. Therefore, we calculated the standardized regression coefficients (beta weights) so that the effect of both patient-related factors and the metabolizer status on the platelet reactivity could be compared, giving a crude indication of the relative importance of the different variables (Table 4). This indicates that the variables with the strongest effect on platelet reactivity measured by LTA are metabolizer group (beta weight: 0.227), hemoglobin (beta weight: 0.155), and aspirin use (beta weight: −0.139). For VerifyNow, the variables with the strongest effect on platelet reactivity are hemoglobin (beta weight: −0.337), platelet count (beta weight: −0.268), and metabolizer group (beta weight: 0.212), respectively. However, for platelet reactivity as measured by Multiplate, the metabolizer group status (beta weight: 0.110) is not among the most important variables, which are platelet count (beta weight: 0.265), previous stroke (beta weight: 0.163), and concomitant use of (es-) omeprazole (beta weight 0.156).

## 4. Discussion

In this study, we investigated the differential impact of *CYP2C19* allelic variants on ADP-induced platelet aggregation as measured by three different platelet function tests in high-risk patients on clopidogrel. According to the Clinical Pharmacogenetics Implementation Consortium (CPIC) guidelines, patients in our study were divided into four metabolizer groups: poor, intermediate, extensive, and rapid metabolizers. The distribution of patients within these metabolizer groups is in line with previous reports in a European population [8]. We showed that residual platelet reactivity as measured by VerifyNow and LTA follows an ordinal trend for the different metabolizer groups, whereas for the Multiplate test, no such trend could be shown, suggesting that the genetic background has little effect on residual platelet reactivity as measured by the Multiplate assay.

Genetic variation in *CYP2C19* is certainly not the only factor that determines the response to clopidogrel. Previous studies have revealed that the CYP2C19*2 polymorphism accounted for only 5–12% of clopidogrel variability in platelet reactivity [6,35]. Other factors, such as body weight, age, health conditions, and concomitant medication, also influence patients’ response to clopidogrel [35]. Although the CYP2C19*2 loss-of-function polymorphism was the strongest predictor of high on-treatment platelet reactivity in the study by Hochholzer et al., the CYP2C9*2 carrier status, together with demographic and clinical predictors for high on-clopidogrel platelet reactivity, could only explain 11.5% of residual platelet reactivity in this study [35]. This indicates that there are still some relevant factors interfering with clopidogrel response that are yet unknown. These known and unknown specific patient-related factors have a differential influence on the results of ex vivo PFTs [36]. Our data suggest that in the Multiplate test, these patient-related factors might prevail and that genetic background only plays a minor role. Comparing the beta weights of the different variables, we showed indeed that the influence of factors such as platelet count, previous stroke, and concomitant medication is more pronounced in the Multiplate test, whereas metabolizer status had less effect on platelet reactivity as measured by this assay. In a previous study, we already showed that the agreement between various PFTs is only slight to moderate and that PFTs may be affected by different factors to a variable degree [24]. The current study adds the underlying *CYP2C19* allelic variation as one of these factors that might further explain this disagreement between platelet function tests.

Another factor to consider when interpreting our findings is the differences between test principles. Multiplate and VerifyNow are whole-blood tests, whereas in LTA, platelet aggregation is assessed in platelet-rich plasma. Moreover, the assays differ in anticoagulant used in the test tubes. Multiplate is performed in hirudin-anticoagulated blood, compared to citrated blood for LTA and VerifyNow. The low calcium environment in citrated blood compared to hirudin blood could attenuate platelet aggregation [37]. Furthermore, the VerifyNow test includes prostaglandin E1 to suppress the platelet activation contribution of ADP binding to the P2Y1 receptor (which is unaffected by clopidogrel administration), and thereby, the assay selectively measures the ADP-P2Y12 pathway [38,39]. VerifyNow is an aggregation-based test using fibrinogen-coated beads, whereas LTA depends completely on the aggregation of platelets in the plasma environment. Lastly, the Multiplate test is based on the measurement of the increase in electrical impedance when platelets adhere and aggregate on two silver-coated copper wires. Thus, aggregation in Multiplate and VerifyNow takes place on surfaces, whereas in LTA, aggregation occurs more or less in a liquid phase [40].

Our findings are in concordance with Harmsze et al., who evaluated the impact of genotypes on on-treatment platelet reactivity as measured by LTA and VerifyNow [41]. Similar to our results, they found for both assays a decreasing trend in residual platelet reactivity comparing PM, IM, EM, and RM groups. However, in this study, the Multiplate assay was not evaluated. Another study that found differences in correlation with genetic background between two different platelet function assays studied the effect of CYP2C19*17 in 598 ACS patients after loading dose and observed a significant impact of the *17 carriage on clopidogrel responsiveness when measured with vasodilator-stimulated phosphoprotein (VASP) assay, but not with LTA measurements [42]. Platelet response in LTA was assessed using 10 μmol ADP compared to 20 μmol ADP in our assay, and, contrary to our study, they did not evaluate the loss-of-function allelic variants and did not compare different metabolizer groups. Finally, Gremmel et al. investigated the influence of CYP2C9 allelic variants on ADP-induced platelet aggregation was determined by five different PFTs, including LTA, Multiplate, and VerifyNow. Although investigating the differential impact of CYP2C9 allelic variants instead of *CYP2C19*, their findings were comparable to ours; a significantly higher platelet reactivity was found for patients with loss-of-function status compared to the normal-function genotype using the VerifyNow assay or LTA, while results did not differ for the Multiplate assay [43].

Recently, several studies evaluated pharmacogenomic testing as an approach of personalized antiplatelet drug administration by either escalation or de-escalation of P2Y12 inhibitor therapy based on *CYP2C19* allelic variants [29,30,44,45]. The POPular Genetics trial demonstrated that a personalized approach using genetic testing to de-escalate to clopidogrel was noninferior to standard treatment with either ticagrelor or prasugrel in terms of the primary composite outcome net clinical benefit (consisting of all-cause death, recurrent MI, definite stent thrombosis, stroke, and PLATO major bleeding), while it was superior in reducing combined major and minor bleedings [29]. A meta-analysis of eight randomized controlled trials, including the recently published TAILOR-PCI trial [30], confirmed that in patients with *CYP2C19*, loss-of function allele prescription of ticagrelor and prasugrel compared to clopidogrel resulted in a significant reduction in ischemic events (RR: 0.70; 95% CI: 0.59–0.83) but not in noncarriers of these alleles (RR: 1.0; 95% CI: 0.80–1.25) [32].

Current guidelines recommend risk stratification for tailoring individual treatment strategies [46,47]. Both platelet function testing and genotyping can provide useful prognostic insights, but trials evaluating treatment strategies have produced mixed results. Pharmacogenomic testing could be an attractive approach because treatment decisions can be made before the start of antiplatelet therapy, unlike with PFT, and the genotype does not change over time, unlike the phenotype of platelet reactivity [48,49]. One of the major limitations of PFTs is the great variability of the results, as is also shown in our study, and this could be overcome by genetic testing. On the other hand, the information derived from genotyping cannot be taken as a surrogate for PFT to assess antiplatelet drug responses, as genetic variants are just one influential factor affecting clopidogrel activity, and numerous epigenetic factors such as comorbidities, gastrointestinal absorption, drug interactions, and adherence are also important determinants. Thus, insight into the relationship between PFT results and genetics, as provided by this study, remains useful in further optimization of antiplatelet strategies. Furthermore, our findings could be of value to laboratories without the opportunity of performing genetic tests or that have to make a choice in the multitude of PFTs.

Important strengths of our study include the careful evaluation of included patients and the concomitant comparison of three different PFTs. Tests were performed 1–2 months after PCI, when a stable situation had been reached, not only in the inflammatory response to stent placement but also in individual clopidogrel response, and an influence of time from clopidogrel loading to platelet function testing could be excluded. A limitation of this study is that we did not assess plasma levels of the active metabolite of clopidogrel, which could have provided more mechanistic insight into the observed platelet response and correlation with the genetic background.

## 5. Conclusions and Future Remarks

In the near future, studies will keep focusing on the role of platelet function testing and genotyping to guide decision making in (high-risk) patients on antiplatelet therapy. In this study, we have shown that the disagreement between PFTs is partly explained by differences in correlation with genetic background. For Multiplate, no major effect of genetic background could be shown, whereas for VerifyNow and LTA, the residual platelet reactivity in patients treated with clopidogrel correlates well with the underlying *CYP2C19* polymorphism. Understanding the (dis-) agreement between these different PFTs and how this relates to genetic variation in *CYP2C19* will help us in the interpretation of these future clinical trials focusing on personalizing antiplatelet therapy.

## Figures and Tables

**Table 1:** Baseline characteristics for the different metabolizer groups.

	All Patients (*n* = 308)	RM (*n* = 107)	EM (*n* = 112)	IM (*n* = 80)	PM (*n* = 9)	* p* ^
Age, years	75.2 (8.5)	74.9 (9.6)	74.7 (7.9)	76.5 (7.4)	74.3 (9.4)	0.478
Female	127 (41.2)	47 (43.9)	43 (38.4)	34 (42.5)	3 (33.3)	0.807
BMI, kg/m^2^	27.4 (4.5)	27.8 (4.5)	27.2 (4.6)	27.3 (4.6)	25.6 (3.9)	0.477
Current smoking	41 (13.3)	12 (11.2)	18 (16.5)	11 (14.5)	0 (0.0)	0.429
Index PCI–ACS	172 (55.8)	59 55.1	60 (53.6)	47 (58.8)	6 (66.7)	0.809
Index PCI–Elective	136 (44.2)	48 (44.9)	52 (46.4)	33 (41.2)	3 (33.3)	
**Medication**						
Clopidogrel	308 (100.0)	107 (100.0)	112(100.0)	80(100.0)	9 (100.0)	1.000
Aspirin	214 (69.5)	71 (66.4)	77 (68.8)	57 (71.3)	9 (100.0)	0.204
VKA	72 (23.4)	28 (26.2)	27 (24.1)	17 (21.3)	0 (0.0)	0.328
DOAC	32 (10.4)	10 (9.3)	12 (10.7)	10 (12.5)	0 (0.0)	0.668
(es-)omeprazole use	28 (9.1)	9 (8.4)	13 (11.6)	5 (6.3%)	1 (11.1)	0.646
**Risk factors**						
Age ≥ 75 years	193 (62.7)	65 (60.7)	67 (59.8)	56 (70.0)	5 (55.6)	0.459
Women	127 (41.2)	47 (43.9)	43 (38.3)	34 (43.)	3 (33.3)	0.807
Weight < 60 kg	28 (9.1)	11 (10.3)	10 (8.9)	7 (8.8)	0 (0.0)	0.778
Diabetes mellitus	110 (35.7)	35 (32.7)	39 (34.8)	29 (36.3)	7 (77.8)	0.060
Hypertension	258 (83.8)	94 (87.9)	92 (82.1)	66 (82.5)	6 (66.7)	0.313
Anemia	107 (34.7)	37 (34.6)	39 (34.8)	27 (33.8)	4 (44.4)	0.938
Renal dysfunction	178 (57.8)	52 (48.6)	67 (59.8)	53 (66.3)	6 (66.7)	0.088
Liver failure	0 (0.0)	0 (0.0)	0 (0.0)	0 (0.0)	0 (0.0)	-
Peptic ulcer disease	42 (13.6)	12 (11.2)	15 (13.4)	13 (16.3)	2 (22.2)	0.667
Prior major bleeding	47 (15.3)	21 (19.6)	13 (11.6)	11 (13.8)	2 (22.2)	0.360
Previous stroke	89 (28.9)	31 (29.0)	32 (28.8)	24 (30.0)	2 (22.2)	0.971
Use of NSAIDs	14 (4.5)	4 (3.7)	6 (5.4)	4 (5.0)	0 (0.0)	0.850
Use of SSRIs	15 (4.9)	4 (3.7)	7 (6.3)	4 (5.0)	0 (0.0)	0.748
Triple therapy	34 (11.0)	11 (10.3)	15 (13.4)	7 (8.8)	1 (11.1)	0.772
High-risk PCI	17 (5.5)	9 (8.4)	5 (4.5)	3 (3.8)	0 (0.0)	0.398
**Previous history**						
Prior PCI	113 (36.7)	31 (29.0)	42 (37.5)	35 (43.8)	5 (55.6)	0.118
Prior CABG	69 (22.4)	22 (20.6)	30 (26.8)	16 (20.0)	1 (11.1)	0.520
Atrial fibrillation	90 (29.2)	33 (30.8)	30 (26.8)	27 (33.8)	0 (0.0)	0.174
Active malignancy	16 (5.2)	4 (3.7)	6 f(5.4)	5 (6.3)	1 (11.1)	0.732
**Laboratory test**						
Hemoglobin, mmol/L	8.2 (1.1)	8.3 (1.1)	8.2 (1.0)	8.1 (1.1)	7.9 (1.0)	0.582
Platelet count, 1×10^9^/L	257 (76)	269 (85)	256 (73)	246 (66)	218 (45)	0.080
Creatinine, µmol/L	117 (67)	104 (48)	126 (85)	120 (55)	131 (85)	0.102
MDRD-eGFR, mL/min/1.73 m^2^	56.1 (20.1)	59.9 (18.6)	54.9 (21.6)	52 (19.1)	55.4 (23.1)	0.082

Continuous variables are expressed as mean (standard deviation). Categorical variables are expressed as counts (percentages). ^ *p*-value calculated using either Chi-square test for categorical variables or ANOVA for continuous variables. Abbreviations: RM, rapid metabolizers; EM, extensive metabolizers; IM, intermediate metabolizers; PM, poor metabolizers; BMI, body mass index; PCI, percutaneous coronary intervention; VKA, vitamin K antagonist; DOAC, direct oral anticoagulant; CABG, coronary artery bypass graft; NSAIDs, nonsteroidal anti-inflammatory drugs; SSRIs, selective serotonin reuptake inhibitors; MDRD-eGFR, Modification of Diet in Renal Disease–estimated glomerular filtration rate.

**Table 2:** Distribution of *CYP2C19* polymorphisms in study population.

Metabolism	*CYP2C19* Alleles	Frequency *n* (%), *n* = 308	Group Total *n* (%)
Rapid metabolizer (RM)	*1/*17	91 (29.5)	107 (34.7)
	*17/*17	16 (5.2)	
Extensive metabolizer (EM)	*1/*1	112 (36.4)	112 (36.4)
Intermediate metabolizer (IM)	*1/*2	67 (21.8)	80 (26.0)
	*2/*17	12 (3.9)	
	*1/*3	1 (0.3)	
Poor metabolizer (PM)	*2/*2	9 (2.9)	9 (2.9)

**Table 3:** Mean platelet reactivity as measured by the three platelet function tests, expressed per group of *CYP2C19* metabolism.

Platelet Function Test	*CYP2C19* Metabolism *	Patients, *n*(%) Total *n* = 308	Median [IQR]	Jonckheere -Terpstra Test ^
LTA (*n* = 300), % max aggr	Metabolizer status			<0.0001
	Rapid metabolizer	103 (34.3%)	41.0 (30.0–51.0)	0.02
	Extensive metabolizer	110 (36.7%)	46.0 (36.0–54.0)	Ref.
	Intermediate metabolizer	78 (26.0%)	49.0 (39.8–57.0)	0.03
	Poor metabolizer	9 (3.0%)	48.0 (43.0–66.5)	0.08
VerifyNow (*n* = 304), PRU	Metabolizer status			<0.0001
	Rapid metabolizer	106 (34.9%)	150.0 (89.3–195.0]	0.06
	Extensive metabolizer	110 (36.2%)	152.0 (112.0–217.5]	Ref.
	Intermediate metabolizer	80 (26.3%)	193.5 (144.0–236.5]	<0.01
	Poor metabolizer	8 (2.6%)	247.0 (144.0–236.5]	<0.01
Multiplate (*n* = 305), AU	Metabolizer status			0.10
	Rapid metabolizer	105 (34.4%)	49.0 (37.5–64.0)	*n/a*
	Extensive metabolizer	111 (36.3%)	47.0 (33.0–64.0)	
	Intermediate metabolizer	80 (26.3%)	55.5 (41.0–72.8)	
	Poor metabolizer	9 (3.0%)	54.0 (38.5–68.5)	

Values are expressed as median with interquartile range (IQR). Categorical data are expressed as absolute numbers and percentages (*n* (%)). n/a; not applicable (pairwise comparisons are not performed because the overall test does not show significant differences across groups). * Poor metabolizers have genotype CYP2C19*2/*2, intermediate metabolizers CYP2C19*1/*2, *1/*3, or *2/*17, extensive metabolizers CYP2C19*1/*1, and rapid metabolizers CYP2C19*1/*17 or *17/*17. ^ Jonckheere–Terpstra test for ordered alternatives, with pairwise comparisons for the different metabolizer groups compared to extensive metabolizers (reference category).

**Table 4:** Standardized regression coefficients (beta weights) of the multivariable model indicating the relative importance of the different variables on platelet reactivity as measured by the platelet function tests.

Variables	LTA	VerifyNow	Multiplate
	Beta Weight	Beta Weight	Beta Weight
Age > 75 years	0.119	0.077	0.079
Body weight < 60 kg	−0.099	−0.119	−0.068
Diabetes	0.003	0.049	0.054
Renal dysfunction	−0.029	0.053	0.090
Previous stroke	0.112	0.111	0.163 *
Current smoking	−0.054	−0.073	−0.002
(es-) omeprazole use	0.035	0.156	0.156 *
Aspirin use	−0.139 *	0.005	−0.013
Anticoagulant use	0.073	−0.033	0.049
Hemoglobin	0.155 *	−0.337 *	−0.046
Platelet count	−0.072	−0.268 *	0.265 *
Metabolizer group	0.227 *	0.212 *	0.110

* Indicates the 3 variables with the highest beta weights per platelet function test.

## Data Availability

The data presented in this study are available on reasonable request from the corresponding author.

## Appendix A

**Table A1:** Inclusion and exclusion criteria.

**Inclusion Criteria**	**Definition**
PCI 30–90 days before study inclusion	Elective or emergency procedure
Dual/triple antithrombotic therapy	Including clopidogrel as P2Y12 inhibitor
Classified as ‘vulnerable’ by ≥ 3 predefined risk factors:	Age ≥ 75 years Female gender Renal dysfunction (MDRD-eGFR ≤ 60 mL/min) Body weight ≤ 60 kg Hypertension (previously diagnosed, or on medication) Diabetes mellitus Anemia (Hb < 8.2 mmol/L for men, <7.3 mmol/L for women) Previous stroke Previous major bleeding Liver dysfunction (known hepatitis or transplant) History of gastric/duodenal ulcers Daily use of NSAIDs or SSRIs Triple antithrombotic therapy (DAPT + oral anticoagulants) Previous in-stent thrombosis or high-risk coronary stent (e.g., last remaining vessel or left main coronary artery)
*CYP2C19* polymorphism available	Analysis of *CYP2C19* polymorphism performed by June 2018
	
**Exclusion criteria**	**Definition**
Known platelet function disorders	Previously diagnosed platelet function disorders
Recent coronary intervention	PCI or CABG ≤ 7 days
Recent new ischemic event	ACS or stroke ≤ 7 days
Signs of active infection	Fever, antibiotic treatment or hospital admission during laboratory assessment of platelet function
Medication noncompliance	Confirmed noncompliance in antithrombotic medication by patient interview or pharmacy dispensing

Abbreviations: PCI, percutaneous coronary intervention; MDRD-eGFR, Modification of Diet in Renal Disease–estimated glomerular filtration rate; Hb, hemoglobin; NSAIDs, nonsteroidal anti-inflammatory drugs; SSRIs, selective serotonin reuptake inhibitors; DAPT, dual antiplatelet therapy; CABG, coronary artery bypass graft; ACS, acute coronary syndrome.

**Table A2:** Partial regression coefficients of variables for LTA, VerifyNow, and Multiplate in univariate and multivariate analysis.

Variables	LTA ADP20 (*n* = 300)						VerifyNow P2Y12 (*n* = 304)						Multiplate (*n* = 305)					
	Univariate			Multivariate *			Univariate			Multivariate *			Univariate			Multivariate *		
	B	95% CI	*p*	B	95% CI	*p*	B		*p*	B	95% CI	*p*	B	95% CI	*p*	B	95% CI	*p*
Age ≥ 75 years	3.456	−0.072–6.983	0.055	3.746	0.27–7.23	0.035	28.013	11.050–44.976	0.001	11.381	−3.88–26.64	0.143	3.121	−2.373–8.615	0.264	3.493	−1.99–8.97	0.211
Gender, female	−1.292	−4.793–2.209	0.468				3.973	−12.950–20.986	0.644				2.306	−3.097–7.709	0.402			
Weight < 60 kg	−10.356	−16.454–−4.259	0.001	−5.386	−11.64–0.87	0.091	−51.291	−79.540–−23.042	0.000	−30.905	−56.79–−5.02	0.019	−3.585	−13.115–5.944	0.460	−6.025	−15.64–3.59	0.218
Diabetes Mellitus	0.033	−3.537–3.602	0.986	−0.028	−3.45–3.40	0.987	13.874	−3.480–31.229	0.117	7.221	−7.83–22.27	0.346	4.480	−1.044–10.003	0.112	2.753	−2.64–8.15	0.316
Hypertension	−1.171	−5.851–3.510	0.623				4.893	−17.597–27.383	0.669				−1.923	−9.114–5.268	0.599			
Renal dysfunction	−1.263	−4.734–2.209	0.475	−0.878	−4.31–2.56	0.615	25.743	9.140–42.346	0.002	8.096	−7.01–23.20	0.292	4.920	−0.449–10.288	0.072	4.474	−0.96–9.91	0.106
History of bleeding	−0.288	−5.095–4.520	0.906				8.777	−14.269–31.824	0.454				4.724	−2.634–12.082	0.207			
Stroke in history #	4.733	1.013–8.453	0.013	3.699	0.11– 7.29	0.043	17.203	−0.919–35.325	0.063	17.793	2.02–33.57	0.027	8.439	2.641–14.236	0.004	8.390	2.70–14.08	0.004
Current smoking #	−3.147	−8.176–1.883	0.219	−2.370	−7.23–2.49	0.338	−33.738	−58.169–−9.308	0.007	−15.310	−36.84–6.22	0.163	−0.760	−8.635–7.116	0.850	−0.019	−7.65–7.62	0.996
(Es-) omeprazole	1.490	−3.505–6.484	0.558	1.644	−3.97 –7.26	0.565	24.248	0.236–48.259	0.048	39.726	14.96–64.49	0.002	8.702	1.036–16.369	0.026	13.40	4.49–22.32	0.003
Aspirin	−6.837	−10.478–−3.197	0.000	−4.471	−11.64–2.70	0.220	6.074	−12.011–24.160	0.509	−0.286	−31.20–30.63	0.986	−2.491	−8.305–3.324	0.400	−0.979	−12.12–10.17	0.863
Anticoagulant	6.134	2.587–9.681	0.001	2.343	−4.61–9.30	0.508	−3.139	−20.674–14.396	0.725	−4.957	−34.91–24.99	0.745	2.204	−3.422–7.831	0.441	2.064	−8.70–12.83	0.706
Hemoglobin (mmol/L)	2.347	0.774–3.920	0.004	2.145	0.50–3.79	0.011	−20.674	−28.101–−13.248	0.000	−22.703	−30.01–−15.40	0.000	−3.396	−5.864–−0.928	0.007	−1.021	−3.64–1.60	0.444
Platelets (1×10^9^/L)	−0.035	−0.056–−0.014	0.001	−0.013	−0.04–0.01	0.246	−0.223	−0.324–−0.122	0.000	−0.243	−0.34–−0.15	0.000	0.070	0.038–0.103	0.000	0.076	0.04–0.11	0.000
Metabolizer status PM IM EM RM	6.895	−3.222–17.012	0.181	8.644	−1.16–18.44	0.084	79.859	28.945–130.773	0.002	47.029	1.49–92.56	0.043	3.859	−12.154–19.872	0.636	6.243	−9.30–21.78	0.430
	3.173	−1.147–7.492	0.149	2.958	−1.23–7.14	0.165	30.234	9.804–50.664	0.004	23.981	5.64–42.32	0.011	8.264	1.488–15.040	0.017	8.792	2.21–15.37	0.009
	ref	ref	-	ref	ref	-	ref		-	ref	ref	-	ref		-	ref	ref	-
	−4.153	−8.154–−0.151	0.042	−4.648	−8.49–−0.81	0.018	−17.381	−36.306–1.543	0.072	−12.047	−28.87–4.77	0.160	1.757	−4.533–8.047	0.583	1.516	−4.55–7.58	0.623

* Based on existing literature, or if univariate analysis indicated the variable to be associated with at least one of the platelet function tests at *p* < 0.20, this variable was included in the multivariate model. The multivariate model is adjusted for age, weight, diabetes, renal dysfunction, previous stroke, current smoking, use of (es-)omeprazole, aspirin or anticoagulant use, Hb, platelet count, and metabolizer status. # Missing data; current smoking missing *n* = 7, and stroke in history missing *n* = 1. Abbreviations: PM; poor metabolizer, IM; intermediate metabolizer, EM; extensive metabolizer, RM; rapid metabolizer.
